# Efficacy and survival analysis of nimotuzumab combined with concurrent chemoradiotherapy in the treatment of locally advanced nasopharyngeal carcinoma

**DOI:** 10.3389/fonc.2023.1129649

**Published:** 2023-02-06

**Authors:** Lili Wang, Hengzhao Zhuang, Xiaoyan Xu, Juying Zhou, Yang Jiao

**Affiliations:** ^1^ Department of Radiation Oncology, The First Affiliated Hospital of Soochow University, Suzhou, China; ^2^ School of Radiation Medicine and Protection, Medical College of Soochow University, Suzhou, China

**Keywords:** nasopharyngeal carcinoma, nimotuzumab, concurrent chemoradiotherapy, survival outcome, propensity score matching

## Abstract

**Objective:**

This study investigated the curative effect of adding nimotuzumab (NTZ) in patients with locally advanced nasopharyngeal carcinoma (NPC) who were treated with concurrent chemoradiotherapy (CCRT) and explored significant prognostic factors of NPC.

**Materials and methods:**

The clinical data of 307 patients with NPC treated in the First Affiliated Hospital of Soochow University from January 2013 to December 2018 were retrospectively analyzed. The patients were divided into the NTZ-CCRT group and the CCRT group according to whether they were associated with NTZ. We applied propensity score matching to reduce the interference of biases and compared the short-term efficacy and long-term survival rate of the two groups. Moreover, univariate and multivariate analyses were performed for all patients, and subgroup analysis was used to compare the efficacy of therapy combined with NTZ in different subgroups.

**Results:**

In primary nasopharyngeal tumors, the objective response rates in the NTZ-CCRT group and CCRT group were 95.8% and 85.7%, respectively (P =0.007). In cervical positive lymph nodes, the objective response rates in the NTZ-CCRT group and CCRT group were 98.3% and 87.4%, respectively (P =0.001). Compared with CCRT alone, the addition of NTZ significantly improved the 5-year OS (94.1% vs. 81.8%, P=0.014) and the 5-year DFS (84.2% vs. 75.5%, P=0.031) of NPC patients; however, the addition of NTZ was accompanied by more severe hematologic toxicity and acute oral mucositis. Multivariate analysis demonstrated that the addition of NTZ was an important prognostic factor for OS and DFS (HR 0.367, 95% CI 0.167-0.808, P=0.013 for OS and HR 0.536, 95% CI 0.312-0.919, P=0.023 for DFS) and the level of pretreatment LDH (HR 5.170, 95% CI 2.125-12.580, P<0.001 for OS and HR 2.421, 95% CI 1.027-5.707, P=0.043 for DFS). Moreover, patients with high levels of hsCRP before treatment (HR 0.389, 95% CI 0.177-0.853, P=0.018) may gain more benefits from combined treatment with NTZ.

**Conclusions:**

For locally advanced NPC patients treated with concurrent chemoradiotherapy, the addition of NTZ can significantly improve their survival outcome. However, it is necessary to guard against the associated increase in hematological toxicity and acute oral mucositis.

## 1 Introduction

Nasopharyngeal carcinoma (NPC) is one of the most common malignant tumors of the head and neck, and its incidence shows obvious regional aggregation. Cases of NPC are the most prevalent in Southeast Asia and southern China (1). Due to the complexity of the anatomical location and the sensitivity of radiation, radiotherapy is often the first choice for treating nasopharyngeal cancer. At present, chemoradiotherapy has become a standardized approach to treat locally advanced nasopharyngeal carcinoma. With the development of radiation technology, including two-dimensional conventional radiotherapy and intensity-modulated radiotherapy(IMRT), the survival time and quality of life of NPC patients have substantially improved (2–4); however, 20% of patients still experience recurrence or metastasis (5). Therefore, it is necessary to explore effective treatment methods, control local recurrence and progression, and improve the survival of NPC patients.

Currently, molecular targeted tumor therapy is an emerging treatment mode of tumor therapy. In nasopharyngeal cancer treatment, the epidermal growth factor receptor (EGFR) is frequently targeted. A study showed that EGFR is highly expressed in 80%-90% of nasopharyngeal cancers (6). After combining with its receptor, EGFR can phosphorylate and activate downstream signaling pathways to regulate the invasion and migration of tumor cells (7). Nimotuzumab (NTZ) is a humanized anti-EGFR monoclonal antibody that can block the EGFR on cells and enhance the radiation sensitivity of tumor cells, thus improving the radiotherapy effect on tumors (8). A retrospective study found that the combination of an anti-EGFR-targeted drug and IMRT was no worse than concurrent chemoradiotherapy (CCRT) in terms of survival outcomes, and relatively few cases of hematological toxicity and gastrointestinal reactions were observed (9). This study retrospectively analyzed the clinical data of patients with locally advanced nasopharyngeal carcinoma who received nimotuzumab combined with concurrent chemoradiotherapy in the First Affiliated Hospital of Soochow University from January 2013 to December 2018; we compared the efficacy and safety of treatment and investigated the prognostic factors, such as pretreatment LDH and pretreatment hsCRP, affecting the survival of patients with nasopharyngeal carcinoma.

## 2 Materials and methods

### 2.1 Patient selection and information collection

A total of 307 patients with locally advanced nasopharyngeal carcinoma who received initial treatment at the First Affiliated Hospital of Soochow University from January 2013 to December 2018 were selected. The assessment of the stage of disease was performed according to the American Joint Committee on Cancer–Union for International Cancer Control 7th edition stage-classification system (10). The inclusion criteria were as follows: (1) nasopharyngeal carcinoma diagnosed by histology and pathology; (2) good general condition, ECOG score 0-2; (3) stage II to IVA; and (4) nimotuzumab targeted therapy. The exclusion criteria included the following: (1) suffering from other malignancies; (2) expected survival less than 6 months; (3) receiving targeted drugs other than anti-EGFR; and (4) incomplete clinical data. Patients were divided into the study group (NTZ-CCRT) and the control group (CCRT) according to whether they were treated with NTZ.

### 2.2 Treatment

#### 2.2.1 Radiotherapy

All patients received IMRT. The GTVnx was defined as gross tumor observed by MR/CT and other images and physical or endoscopic examination, while the GTVnd was defined as highly suspicious lymph nodes with a short diameter over 1 cm, necrosis, and increased glucose metabolism observed by FDG-PET. CTV1, the high-risk clinical target volume, was defined as the GTVnx plus a 5–10 mm margin (2–3 mm posteriorly) to encompass the high-risk sites of microscopic extension and the whole nasopharynx. The CTV2 included the CTV1 plus a 5–10 mm margin (2–3 mm posteriorly) and the cervical level where the positive lymph nodes were located. The CTV3 was defined as the low-risk cervical area with no positive lymph nodes. Prescription dose: PGTVnx: 68-70 Gy, PGTVnd: 68 Gy, PCTV1: 66 Gy, PCTV2: 64 Gy, PCTV3: 54-60 Gy, 31-33 fractions. All patients were treated once daily with five fractions every week.

#### 2.2.2 Chemotherapy

Patients in both groups received 1-2 cycles of induction chemotherapy before radiotherapy and 1-2 cycles of concurrent chemotherapy during radiotherapy. The regimen consisted of taxane or fluorouracil plus cisplatin or nedaplatin every 3 weeks. After radiotherapy, 0-4 cycles of adjuvant chemotherapy were administered according to the tumor regression.

#### 2.2.3 Targeted therapy

Based on the treatment in the control group (CCRT), nimotuzumab (50 mg/10 ml, Baitai Biology Pharmaceutical Co., Ltd.) was used in the study group (NTZ-CCRT). During radiotherapy, the 200 mg dose was diluted with 250 ml of normal saline once per week, and the duration of intravenous injection was not less than 60 min.

### 2.3 Follow-up and end-points

Follow-up was mainly carried out through a combination of medical record review and telephone contacts. The deadline for the last follow-up was March 1, 2022. The range of follow-up was 8-110 months, with a median follow-up of 57 months. The primary endpoints of this study were the objective response rate (ORR) and disease control rate (DCR). The efficacy evaluation criteria were based on the Response Evaluation Criteria in Solid Tumors (RECIST version 1.1), divided into complete response (CR), partial response (PR), stable disease (SD) and progressive disease (PD), in which CR+PR indicated efficacy and CR+PR+SD represented adequate control. The secondary endpoints included overall survival (OS), disease-free survival (DFS), distant metastasis-free survival (DMFS), and local recurrence-free survival (LRRFS). OS was defined as the time until death from any cause, and DFS was defined as the time until the date of death or first metastasis, progression, or recurrence, whichever occurred first. DMFS was defined as the time until the first distant metastasis, and LRRFS was defined as the time until the first local recurrence. Chemotherapy-related adverse reactions were evaluated according to the Common Terminology Criteria for Adverse Events (CTCAE version 5.0), skin and mucosal reactions were evaluated according to the toxicity criteria of the Radiation Therapy Oncology Group (RTOG) (11), and the maximum value of toxicity was recorded and assessed.

### 2.4 Statistical analysis

Propensity score matching (PSM) was used to adjust the age, sex, T stage, N stage and other factors of the two groups, and 1:1 nearest neighbor matching was conducted to create a well-balanced cohort for the comparison of effectiveness and safety. SPSS software (version 25.0) was used for data processing, and a P value less than 0.05 was considered statistically significant. The χ2 test and the corrected χ2 test or Fisher’s exact test were used for categorical variable comparisons, and the independent sample T test or Mann−Whitney U test was used for continuous variable comparisons. The survival results were calculated by the Kaplan−Meier method, and the survival differences between groups were compared by the log-rank test. A univariate analysis was performed for all eligible cases, and the potentially significant variables included sex, age, T stage, N stage, pretreatment LDH, pretreatment hsCRP, etc. To explore the independent prognostic factors of survival, the factors that conformed to the conditions of equal proportion hypothesis risk were included in the multivariate Cox regression model. In addition, we used a subgroup analysis method to compare the efficacy of chemoradiotherapy combined with NTZ in different subgroups.

## 3 Results

### 3.1 Baseline characteristics

A total of 307 eligible patients with locally advanced nasopharyngeal carcinoma were enrolled, including 241 males (78.5%) and 66 females (21.5%). The median age of the patients was 53 years. We used propensity score matching to create a well-balanced cohort of 238 patients, with 119 patients in the NTZ-CCRT group and 119 patients in the CCRT group. After propensity score matching, the sex, age, T stage, N stage and other variables of the two groups were not statistically significant, as shown in **Table 1**.

**Table 1:** Characteristics of NPC patients treated with or without nimotuzumab.

Characteristics	NTZ-CCRT group (n =119)	CCRT group (n =119)	P value
Age			0.882
**Median**	52	53	
**Range**	26-73	22-74	
Gender (%)			0.750
**Male**	**95 (79.8)**	**93 (78.2)**	
**Female**	**24 (20.2)**	**26 (21.8)**	
T-stage (%)			1.000
**T1-2**	**76 (63.9)**	**76 (63.9)**	
**T3-4**	**43 (36.1)**	**43 (36.1)**	
N-stage (%)			0.599
**N0-1**	**18 (15.1)**	**21 (17.6)**	
**N2-3**	**101 (84.9)**	**98 (82.4)**	
HGB (g/L)			0.500
**Median**	145	145	
**Range**	90-173	109-187	
Albumin (g/L)			0.743
**Median**	45.3	45.5	
**Range**	35.3-54.3	36.3-52.2	
hsCRP (mg/L)			0.299
**Median**	1.3	3.53	
**Range**	0.1-23.6	0.03-28.90	
LDH (U/L)			0.629
**Median**	176.6	177.0	
**Range**	109.0-310.0	119.0-420.8	
BMI (kg/m2)			0.637
**Median**	23.53	23.53	
**Range**	17.43-31.63	17.48-32.34	
Histological classification			0.651
**undifferentiation**	116 (97.5%)	117 (98.3%)	
**differentiation**	3 (2.5%)	2 (1.7%)	
Chemotherapy			0.247
**TP**	119	116	
**FP**	0	3	

NTZ-CCRT, Nimotuzumab-Concurrent Chemoradiotherapy; CCRT, Concurrent Chemoradiotherapy; HGB, Hemoglobin hs; CRP, High Sensitivity-C reactive protein; LDH, Lactate Dehydrogenase; BMI, Body Mass Index; TP, Taxane plus Platinum; FP, Fluorouracil plus Platinum.

### 3.2 Efficacy

After propensity score matching, a total of 238 cases were evaluated for short-term efficacy. For primary nasopharyngeal tumors, the ORRs of the NTZ-CCRT group and CCRT group were 95.8% and 85.7%, respectively (P =0.007), and the DCR was 100%. Specifically, in the NTZ-CCRT group, we observed the following: CR 48.7% (58/119), PR 47.1% (56/119), SD 4.2% (5/119), and PD 0% (0/119). In the CCRT group, we observed the following: CR 47.9% (57/119), PR 37.8% (45/119), SD 14.3% (17/119), and PD 0% (0/119). In the cervical positive lymph nodes, the objective response rates in the NTZ-CCRT group and CCRT group were 98.3% and 87.4%, P =0.001, and the DCR was 100%. In the NTZ-CCRT group, we observed the following: CR 48.7% (56/115), PR 49.6% (57/115), SD 1.7% (2/115), and PD 0% (0/115). In the CCRT group, we observed the following: CR 49.6% (59/119), PR 37.8% (45/119), SD 12.6% (15/119), and PD 0% (0/119). These results are shown in **Tables 2**, **3**.

**Table 2:** Comparison of short-term efficacy in nasopharyngeal tumor patients.

Groups	N	CR	PR	SD	PD	ORR(%)	DCR(%)
**NTZ-CCRT group**	119	58 (48.7)	56 (47.1)	5 (4.2)	0 (0.0)	114 (95.8)	119 (100.0)
**CCRT group**	119	57 (47.9)	45 (37.8)	17 (14.3)	0 (0.0)	102 (85.7)	119 (100.0)
***χ^2^* value**						7.212	
***P* value**						0.007	

CR, Complete Response; PR, Partial Response; SD, Stable Disease; PD, Progressive Disease; ORR, Objective Response Rate; DCR, Disease Control Rate.

**Table 3:** Comparison of short-term efficacy in cervical positive lymph nodes patients.

Groups	N	CR	PR	SD	PD	ORR(%)	DCR(%)
**NTZ-CCRT group**	115	56 (48.7)	57 (49.6)	2 (1.7)	0 (0.0)	113 (98.3)	115 (100.0)
**CCRT group**	119	59 (49.6)	45 (37.8)	15 (12.6)	0 (0.0)	104 (87.4)	119 (100.0)
***χ^2^* value**						10.249	
***P* value**						0.001	

### 3.3 Survival

**Figure 1:**
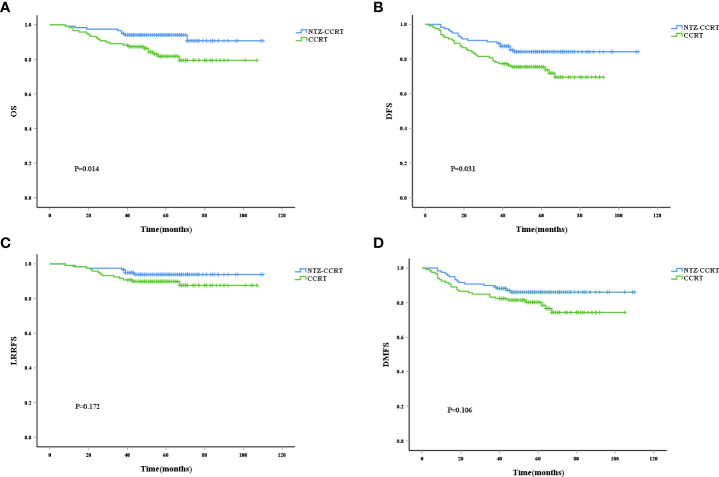
Kaplan-Meier curves of overall survival **(A)**, disease-free survival **(B)**, local recurrence-free survival **(C)**, and distant metastasis-free survival **(D)**.

In the entire cohort, 26 patients had local recurrence, 56 patients had distant metastasis, and 39 patients died from tumor-related causes. After PSM, the 5-year OS rates in the NTZ-CCRT group and CCRT group were 94.1% vs. 81.8%, respectively (P=0.014) (**Figure 1A**), and the difference was statistically significant. In terms of the DFS, the 5-year DFS rates in the NTZ-CCRT group and CCRT group were 84.2% and 75.5%, P=0.031 (**Figure 1B**), respectively. The 5-year LRRFS rates in the NTZ-CCRT group and CCRT group were 93.9% and 89.9%, P=0.172 (**Figure 1C**), respectively, with no statistically significant difference. In regard to the 5-year DMFS, there were no statistically significant differences between the NTZ-CCRT group and the CCRT group (86.1% vs. 80.3%, P=0.106) (**Figure 1D**).

### 3.4 Safety

The most common adverse reactions in the NTZ-CCRT group were mucositis, leukopenia, and anemia; the most common grade 3/4 adverse reactions were mucositis, leukopenia, and neutropenia; the most common adverse reactions in the CCRT group mainly included mucositis, anemia, and leukopenia; and the most common grade 3/4 adverse reactions included mucositis, leukopenia, and neutropenia, as shown in **Table 4**. Compared with that in the CCRT group, the incidence of hematological toxicity was higher in the NTZ-CCRT group, primarily manifested leukopenia and neutropenia, and there was no significant difference in the incidence of hepatic toxicity. In addition, more patients in the NTZ-CCRT group suffered from mucositis than those in the CCRT group (103/119 in the NTZ-CCRT group and 81/119 in the CCRT group), and the incidence rates of mucositis were 86.5% vs. 68.1% (P=0.001), respectively. There were also more patients who suffered grade 3/4 mucositis in the NTZ-CCRT group than in the CCRT group (49.6% vs. 37.8%, P=0.067), although the difference was not statistically significant.

**Table 4:** Acute toxicities in the NPC patients.

Adverse events	NTZ-CCRT			CCRT			χ^2^	P
	0	1/2	3/4	0	1/2	3/4		
**Leukopenia**	22	61	36	43	43	33	9.334	0.002
**Neutropenia**	57	36	26	77	21	21	6.831	0.009
**Anemia**	28	90	1	40	74	5	2.965	0.085
**Thrombocytopenia**	80	37	2	76	41	2	0.298	0.585
**ALT increased**	105	13	1	113	5	1	3.494	0.062
**AST increased**	112	6	1	113	6	0	0.081	0.775
**Skin reaction**	109	6	4	113	3	3	1.072	0.300
**Mucositis**	16	44	59	38	36	45	11.59	0.001

### 3.5 Univariate and multivariate analysis

First, we performed univariate analysis using data for the entire cohort. The results are summarized in **Table S1**. The potential prognostic factors for OS included combination with NTZ, sex, age, and LDH level before treatment. The potential prognostic factors for DFS included combination with NTZ and sex, while the combination with NTZ, age and T-stage were potential prognostic factors for DMFS. According to the results of the univariate analysis, we included NTZ, sex, age, LDH and hsCRP before treatment in the Cox multivariate regression model. In addition, we also conducted correlation analyses for T stage and N stage, and there was no correlation among them (P=0.366). Therefore, the T stage and N stage variables were both included in the multifactor analysis. The above factors all meet the conditions of the equal proportion hypothesis risk. The results of the multivariate analysis are shown in **Table S2**. The addition of NTZ (HR 0.367, 95% CI 0.167-0.808, P=0.013), sex (HR 0.155, 95% CI 0.037-0.650, P=0.011), and pretreatment LDH level (HR 5.170, 95% CI 2.125-12.580, P < 0.001) were important prognostic factors for OS, and the addition of NTZ (HR 0.536, 95% CI 0.312-0.919, P=0.023), sex (HR 0.440, 95% CI 0.207-0.931, P=0.032), T stage (HR 1.734, 95% CI 1.065-2.825, P=0.027) and pretreatment LDH level (HR 2.421, 95% CI 1.027-5.707, P=0.043) were important prognostic factors for DFS. The important prognostic factors for DMFS were age (HR 1.918, 95% CI 1.098-3.350, P=0.022), T stage (HR 2.000, 95% CI 1.176-3.403, P=0.011), and pretreatment LDH level (HR 2.997, 95% CI 1.260-7.131, P=0.013).

### 3.6 Subgroup analysis

**Figure 2:**
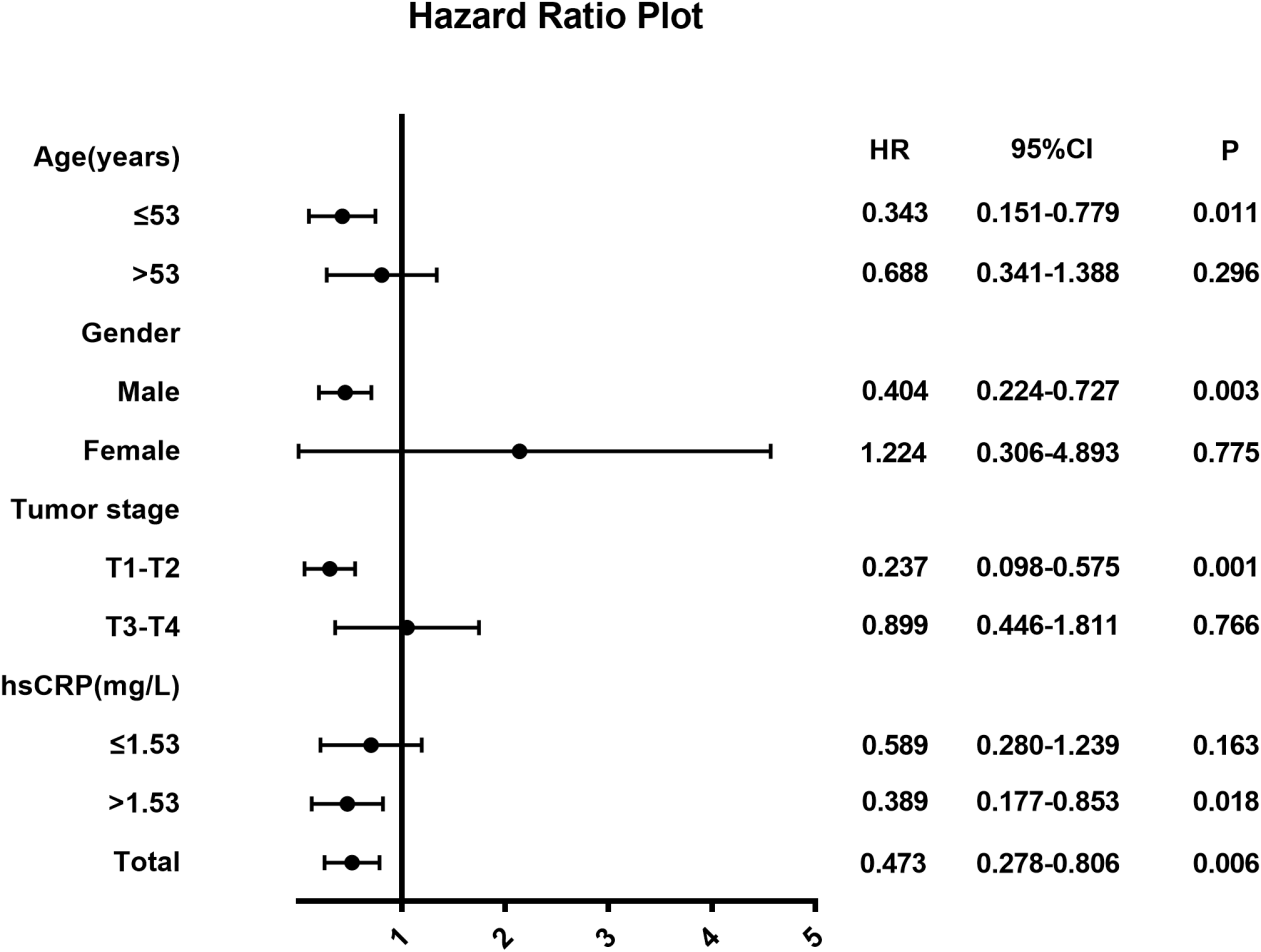
Hazard ratios for death in NPC patients.

We performed a subgroup analysis to explore whether treatment combined with NTZ was beneficial in each subgroup (**Figure 2**). In routine clinical practice, the critical value of LDH before treatment is 250 U/L. The cutoff value of the pretreatment hsCRP was the median hsCRP level in the whole group, which was 1.53 mg/L. The results of the subgroup analysis are shown in **Figure 2**. In terms of DFS, we found that in the T1-T2 (HR 0.237, 95% CI 0.098-0.575, P=0.001), age ≤53 years (HR 0.343, 95% CI 0.151-0.779, P=0.011), and male subgroups (HR 0.404, 95% CI 0.224-0.727, P=0.003), patients treated with NTZ appeared to gain more survival benefits. In addition, different levels of hsCRP before treatment may have affected the efficacy of the addition of NTZ, and the results showed that patients with higher levels of hsCRP before treatment (HR 0.389, 95% CI 0.177-0.853, P=0.018) may gain more benefits from the addition of NTZ.

## 4 Discussion

With tumor treatment entering the era of comprehensive treatment, molecular targeted therapy has attracted widespread attention. The combination of anti-EGFR drugs with radiotherapy and chemotherapy has been widely used in the treatment of a variety of cancers, including nasopharyngeal cancer, colorectal cancer, and non-small cell lung cancer (12–14). Some studies have documented that EGFR is overexpressed in 80%-90% of nasopharyngeal cancers (6), often resulting in poor survival, possibly associated with resistance to radiation or drugs (15). It has been observed that nimotuzumab enhances the radiosensitivity of tumor cells in lung and breast cancer by blocking nuclear translocation in EGFR and inhibiting repair caused by radiation-induced DNA damage (8, 16). Increased radiosensitivity has also been observed in nasopharyngeal carcinoma cells with the use of nimotuzumab (17). A study showed that the binding affinity of nimotuzumab to EGFR is related to the distribution density of EGFR in tissues. In normal tissues with low EGFR density, such as the skin and mucous membrane, the binding force of nimotuzumab is low, resulting in mild cytotoxicity (18). Therefore, nimotuzumab combined with radiotherapy is widely used in the treatment of epithelium-derived tumors.

A multicenter randomized controlled study that was conducted in an area where nasopharyngeal cancer is prevalent showed that in patients with locally advanced nasopharyngeal carcinoma who received cisplatin plus IMRT, the treatment with nimotuzumab combined with PF led to better lymph node response rates than treatment with PF alone (81% vs. 60%, P=0.036), with a statistically significant difference (19). These outcomes are consistent with our study. We found that, in terms of the short-term efficacy of cervical lymph nodes, the NTZ-CCRT group had a better lymph node effect than the CCRT group, with objective remission rates in the two groups of 98.3% vs. 87.4%, respectively (P =0.001). Another phase II trial in patients with N3M0 nasopharyngeal carcinoma found that after induction chemotherapy followed by nimotuzumab combined with CCRT, the effective rate was 100%, and the 3-year local control rates reached 97.8% (20). We found that tumor cells in the cervical metastatic lymph nodes also had high EGFR expression. A study showed that the positive rate of EGFR expression in primary nasopharyngeal carcinoma and metastatic lymph nodes was 73.3% and 60.5%, respectively, and the difference in the expression level between primary and positive lymph nodes was statistically significant (P=0.001) (21). Nimotuzumab binds to the EGFR receptors on tumor cells in cervical metastatic lymph nodes to enhance radiotherapy sensitivity, thereby increasing lymph node response rates and improving local response rates.

Our study found that adding NTZ to concurrent chemoradiotherapy led to better results in patients with nasopharyngeal primary tumors. The ORRs of the NTZ-CCRT group and the CCRT group were 95.8% vs. 85.7%, p=0.007, showing a significant difference. A meta-analysis conducted by Li, which included 9 randomized controlled trials and 6 cohort studies with a total of 1 015 patients, found that combined chemoradiotherapy with nimotuzumab was associated with increased response rates compared to chemoradiotherapy alone (RR =1.11, 95% CI: 1.01-1.22). It has been confirmed that nimotuzumab combined with concurrent chemoradiotherapy is superior to chemoradiotherapy alone in patients with advanced nasopharyngeal carcinoma (22). Regarding the long-term efficacy, we further observed that the combination of NTZ did not seem to have an advantage in terms of relapse-free survival, and the LRRFS of the NTZ-CCRT group and the CCRT group were 97.5% vs. 91.6% (P=0.172). However, in terms of the overall survival and progression-free survival, the addition of NTZ had obvious advantages. The 3-year OS rates in the NTZ-CCRT group and the NTZ group were 96.6% and 89.1%, respectively (P=0.014), and the 3-year DFS rates were 89.9% and 78.2%, respectively (P=0.031). This was also confirmed in the multivariate analysis, and the addition of NTZ was an important prognostic factor for OS (HR 0.367, 95% CI 0.167-0.808, P=0.013). In addition, the addition of NTZ was also an important prognostic factor for DFS (HR 0.536, 95% CI 0.312-0.919, P=0.023), indicating that it may further improve survival by improving DFS. We also observed that the LDH level before treatment was a prognostic factor of OS, DFS and DMFS, and the HRs were 5.170, 2.421 and 2.997, respectively, with P values less than 0.05. The survival time of the NPC patients was correlated with pretreatment LDH levels, and higher LDH levels were associated with poorer survival. Wan also observed an association between pretreatment LDH levels and survival in nasopharyngeal carcinoma patients, with increased LDH levels predicting worse OS (56.9% vs. 76.8%, P =0.004), DFS (45.4% vs. 64.7%, P =0.001), DMFS (54.3% vs. 72.2%, P =0.001) and LRRFS (76.1% vs. 89.6%, P =0.019). Multivariate analysis confirmed that the LDH level before treatment was an independent prognostic factor for patients with locally advanced nasopharyngeal carcinoma (23). A meta-analysis aimed at investigating the prognostic value of pretreatment serum LDH levels in NPC patients among the Chinese population also showed that high LDH levels were significantly associated with poor OS, DFS, and DMFS (24).

A previous study showed that pretreated hsCRP levels are associated with survival in patients with nasopharyngeal cancer. Tang found that hsCRP was connected with OS (HR: 1.723; 95% CI: 1.238-2.398; P=0.001), PFS (HR: 1.621; 95% CI: 1.273-2.064; P<0.001) and DMFS (HR: 1.879; 95% CI: 1.394-2.531; P < 0.001). They concluded that elevated serum hsCRP levels predicted lower survival in NPC patients. It may be complementary to TNM staging and EBV DNA in terms of prediction (25). In the subgroup analysis of DFS, our study found that different levels of pretreatment hsCRP may affect the curative effect of NTZ. In the subgroup with hsCRP > 1.53 mg/L, additional NTZ may have a survival benefit (HR 0.389, 95% CI 0.177-0.853, P=0.018). In addition, we observed that in the T1-T2 subgroup, the addition of NTZ seemed to have better efficacy than in the control group (HR 0.237, 95% CI 0.098-0.575, P=0.001).

In this study, compared with the CCRT group, a higher incidence of adverse reactions was observed in the NTZ-CCRT group, including acute oral mucositis (86.5% vs. 68.1%, P=0.001) and leucopenia (81.5% vs. 63.9%, P=0.002). The incidence of grade 3/4 oral mucositis in the NTZ-CCRT group and CCRT group was 49.6% vs. 37.8%, P=0.067, and no adverse reactions, such as rash, allergy or abnormal renal function, were observed. We analyzed the high incidence of oral mucosal reactions in patients treated with extra NTZ, which may have been related to the reduced repair ability of epithelial cells caused by anti-EGFR drugs. However, with proper care and treatment, a large number of patients can tolerate this treatment.

This was a single-center retrospective study, and although some biases were eliminated by propensity score matching, it is unclear whether there were other biases. Therefore, further multicenter prospective studies are necessary to evaluate the efficacy and safety of nimotuzumab in patients with locally advanced nasopharyngeal carcinoma. In conclusion, compared with chemoradiotherapy alone, patients with locally advanced nasopharyngeal cancer who receive chemoradiotherapy plus nimotuzumab can benefit in terms of short-term efficacy and long-term survival. It is necessary to guard against the increase in hematological toxicity and acute oral mucositis, and pain can be alleviated through good nursing care and supportive treatment.

## Data availability statement

The original contributions presented in the study are included in the article/**Supplementary Material**. Further inquiries can be directed to the corresponding authors.

## Ethics statement

The studies involving human participants were reviewed and approved by the Ethics Committee of the First Affiliated Hospital of Soochow University. The patients/participants provided their written informed consent to participate in this study.

## Author contributions

All authors listed have made a substantial, direct, and intellectual contribution to the work and approved it for publication.

## References

[1] Cao SM Simons MJ Qian CN. The prevalence and prevention of nasopharyngeal carcinoma in China. Chin J Cancer. (2011) 30(2):114–9. doi: 10.5732/cjc.010.10377 PMC4013340 21272443

[2] Zhao W Lei H Zhu X Li L Qu S Liang X. Investigation of long-term survival outcomes and failure patterns of patients with nasopharyngeal carcinoma receiving intensity-modulated radiotherapy: A retrospective analysis. Oncotarget. (2016) 7(52):86914–25. doi: 10.18632/oncotarget.13564 PMC5349963 27894100

[3] You R Cao YS Huang PY Chen L Yang Q Liu YP. The changing therapeutic role of chemo-radiotherapy for loco-regionally advanced nasopharyngeal carcinoma from Two/Three-dimensional radiotherapy to intensity-modulated radiotherapy: A network meta-analysis. Theranostics (2017) 7(19):4825–35. doi: 10.7150/thno.21815 PMC5706102 29187906

[4] Tang LL Chen L Mao YP Li WF Sun Y Liu LZ. Comparison of the treatment outcomes of intensity-modulated radiotherapy and two-dimensional conventional radiotherapy in nasopharyngeal carcinoma patients with parapharyngeal space extension. Radiother Oncol (2015) 116(2)167–73. doi: 10.1016/j.radonc.2015.07.038 26316395

[5] You R Hua YJ Liu YP Yang Q Zhang YN Li JB. Concurrent chemoradiotherapy with or without anti-EGFR-Targeted treatment for stage II-IVb nasopharyngeal carcinoma: Retrospective analysis with a Large cohort and long follow-up. Theranostics (2017) 7(8):2314–24. doi: 10.7150/thno.19710 PMC5505063 28740554

[6] Zhang P Wu SK Wang Y Fan ZX Li CR Feng M. MDM2, eIF4E and EGFR expression in nasopharyngeal carcinoma and their correlation with clinicopathological characteristics and prognosis: A retrospective study. Oncol Lett (2015) 9(1):113–8. doi: 10.3892/ol.2014.2631. p53. PMC4246848 25435943

[7] Xu MJ Johnson DE Grandis JR. EGFR-targeted therapies in the post-genomic era. Cancer Metastasis Rev (2017) 36(3):463–73. doi: 10.1007/s10555-017-9687-8 PMC5693744 28866730

[8] Teng K Zhang Y Hu X Ding Y Gong R Liu L. Nimotuzumab enhances radiation sensitivity of NSCLC H292 cells *in vitro* by blocking epidermal growth factor receptor nuclear translocation and inhibiting radiation-induced DNA damage repair. Onco Targets Ther (2015) 8:809–18. doi: 10.2147/ott.S77283 PMC4403694 25926742

[9] You R Sun R Hua YJ Li CF Li JB Zou X. Cetuximab or nimotuzumab plus intensity-modulated radiotherapy versus cisplatin plus intensity-modulated radiotherapy for stage II-IVb nasopharyngeal carcinoma. Int J Cancer (2017) 141(6):1265–76. doi: 10.1002/ijc.30819 28577306

[10] Edge SB Compton CC. The American joint committee on cancer: The 7th edition of the AJCC cancer staging manual and the future of TNM. Ann Surg Oncol (2010) 17(6):1471–4. doi: 10.1245/s10434-010-0985-4 20180029

[11] Cox JD Stetz J Pajak TF. Toxicity criteria of the radiation therapy oncology group (RTOG) and the European organization for research and treatment of cancer (EORTC). Int J Radiat Oncol Biol Phys (1995) 31(5):1341–6. doi: 10.1016/0360-3016(95)00060-c 7713792

[12] Yuan C Xu XH Chen Z. Combination treatment with antiEGFR monoclonal antibodies in advanced nasopharyngeal carcinoma: A meta-analysis. J buon. (2015) 20(6):1510–7. 26854448

[13] Van Cutsem E Köhne CH Hitre E Zaluski J Chang Chien CR Makhson A. Cetuximab and chemotherapy as initial treatment for metastatic colorectal cancer. N Engl J Med (2009) 360(14):1408–17. doi: 10.1056/NEJMoa0805019 19339720

[14] Zhong WZ Wang Q Mao WM Xu ST Wu L Shen Y. Gefitinib versus vinorelbine plus cisplatin as adjuvant treatment for stage II-IIIA (N1-N2) EGFR-mutant NSCLC (ADJUVANT/CTONG1104): A randomised, open-label, phase 3 study. Lancet Oncol (2018) 19(1):139–48. doi: 10.1016/s1470-2045(17)30729-5 29174310

[15] Ooft ML Braunius WW Heus P Stegeman I van Diest PJ Grolman W. Prognostic significance of the EGFR pathway in nasopharyngeal carcinoma: A systematic review and meta-analysis. biomark Med (2015) 9(10):997–1010. doi: 10.2217/bmm.15.68 26441207

[16] Qu YY Hu SL Xu XY Wang RZ Yu HY Xu JY. Nimotuzumab enhances the radiosensitivity of cancer cells *in vitro* by inhibiting radiation-induced DNA damage repair. PLoS One (2013) 8(8):e70727. doi: 10.1371/journal.pone.0070727 23976954 PMC3745376

[17] Huang J Yuan X Pang Q Zhang H Yu J Yang B. Radiosensitivity enhancement by combined treatment of nimotuzumab and celecoxib on nasopharyngeal carcinoma cells. Drug Des Devel Ther (2018) 12:2223–31. doi: 10.2147/dddt.S163595 PMC6052925 30038488

[18] Garrido G Tikhomirov IA Rabasa A Yang E Gracia E Iznaga N. Bivalent binding by intermediate affinity of nimotuzumab: A contribution to explain antibody clinical profile. Cancer Biol Ther (2011) 11(4):373–82. doi: 10.4161/cbt.11.4.14097 21150278

[19] Lu Y Chen D Liang J Gao J Luo Z Wang R. Administration of nimotuzumab combined with cisplatin plus 5-fluorouracil as induction therapy improves treatment response and tolerance in patients with locally advanced nasopharyngeal carcinoma receiving concurrent radiochemotherapy: A multicenter randomized controlled study. BMC Cancer (2019) 19(1):1262. doi: 10.1186/s12885-019-6459-6 31888551 PMC6937916

[20] Zhang S Huang X Zhou L Wu G Lin J Yang S. An open-label, single-arm phase II clinical study of induction chemotherapy and sequential nimotuzumab combined with concurrent chemoradiotherapy in N3M0 stage nasopharyngeal carcinoma. J buon (2018) 23(6):1656–61. 30610791

[21] Huang Y Lu TX He JH Luo RZ Lin TY. [Expressions of epidermal growth factor receptor in primary nasopharygeal carcinoma and lymph node metastases]. Nan Fang Yi Ke Da Xue Xue Bao. (2009) 29(5):949–51. 19460717

[22] Li Z Li Y Yan S Fu J Zhou Q Huang X. Nimotuzumab combined with concurrent chemoradiotherapy benefits patients with advanced nasopharyngeal carcinoma. Onco Targets Ther (2017) 10:5445–58. doi: 10.2147/ott.S141538 PMC5694200 29180878

[23] Wan XB Wei L Li H Dong M Lin Q Ma XK. High pretreatment serum lactate dehydrogenase level correlates with disease relapse and predicts an inferior outcome in locally advanced nasopharyngeal carcinoma. Eur J Cancer (2013) 49(10):2356–64. doi: 10.1016/j.ejca.2013.03.008 23541571

[24] Zhang M Wei S Su L Lv W Hong J. Prognostic significance of pretreated serum lactate dehydrogenase level in nasopharyngeal carcinoma among Chinese population: A meta-analysis. Med (Baltimore) (2016) 95(35):e4494. doi: 10.1097/md.0000000000004494 PMC5008543 27583859

[25] Tang LQ Hu DP Chen QY Zhang L Lai XP He Y. Elevated high-sensitivity c-reactive protein levels predict decreased survival for nasopharyngeal carcinoma patients in the intensity-modulated radiotherapy era. PLoS One (2015) 10(4):e0122965. doi: 10.1371/journal.pone.0122965 25874450 PMC4395211

