# Supplementary material for: Efficacy and survival analysis of nimotuzumab combined with concurrent chemoradiotherapy in the treatment of locally advanced nasopharyngeal carcinoma
Source: Front Oncol. 2023 Feb 6;13:1129649. doi: 10.3389/fonc.2023.1129649 (PMC9939457; doi:10.3389/fonc.2023.1129649)
Supplement: Supplementary file 1 [file Table_1.docx]

Table S 1 **Univariate Analysis of Prognostic Factors on survival outcomes in** **NPC patients.**

|  | HR | CI (95%) | P value |
| --- | --- | --- | --- |
| **Overall survival** | | | |
| Treatment |  |  |  |
| CCRT | Reference |  |  |
| NTZ-CCRT | 0.31 | 0.143-0.675 | 0.003 |
| Gender |  |  |  |
| Male | Reference |  |  |
| Female | 0.182 | 0.044-0.753 | 0.019 |
| Age |  |  |  |
| ≤53 | Reference |  |  |
| >53 | 1.034 | 1.004-1.066 | 0.027 |
| Tumor stage |  |  |  |
| T1-T2 | Reference |  |  |
| T3-T4 | 1.664 | 0.888-3.119 | 0.112 |
| Node stage |  |  |  |
| N0-N1 | Reference |  |  |
| N2-N3 | 2.321 | 0.715-7.539 | 0.161 |
| LDH |  |  |  |
| ≤250 | Reference |  |  |
| >250 | 3.557 | 1.489-8.495 | 0.004 |
| hsCRP |  |  |  |
| ≤1.53 | Reference |  |  |
| >1.53 | 1.781 | 0.934-3.398 | 0.080 |
| **Disease–free survival** | | | |
| Treatment |  |  |  |
| CCRT | Reference |  |  |
| NTZ-CCRT | 0.473 | 0.278-0.806 | 0.006 |
| Gender |  |  |  |
| Male | Reference |  |  |
| Female | 0.476 | 0.227-0.997 | 0.049 |
| Age |  |  |  |
| ≤53 | Reference |  |  |
| >53 | 1.488 | 0.913-2.425 | 0.111 |
| Tumor stage |  |  |  |
| T1-T2 | Reference |  |  |
| T3-T4 | 1.664 | 0.888-3.119 | 0.112 |
| Node stage |  |  |  |
| N0-N1 | Reference |  |  |
| N2-N3 | 2.470 | 0.993-6.149 | 0.052 |
| LDH |  |  |  |
| ≤250 | Reference |  |  |
| >250 | 1.860 | 0.803-4.308 | 0.147 |
| hsCRP |  |  |  |
| ≤1.53 | Reference |  |  |
| >1.53 | 1.350 | 0.831-2.193 | 0.226 |
| **Distant metastasis-free survival** | | | |
| Treatment |  |  |  |
| CCRT | Reference |  |  |
| NTZ-CCRT | 0.525 | 0.297-0.927 | 0.026 |
| Gender |  |  |  |
| Male | Reference |  |  |
| Female | 0.496 | 0.225-1.096 | 0.083 |
| Age |  |  |  |
| ≤53 | Reference |  |  |
| >53 | 2.016 | 1.166-3.485 | 0.012 |
| Tumor stage |  |  |  |
| T1-T2 | Reference |  |  |
| T3-T4 | 2.092 | 1.237-3.539 | 0.006 |
| Node stage |  |  |  |
| N0-N1 | Reference |  |  |
| N2-N3 | 2.016 | 0.805-5.052 | 0.135 |
| LDH |  |  |  |
| ≤250 | Reference |  |  |
| >250 | 2.246 | 0.962-5.241 | 0.061 |
| hsCRP |  |  |  |
| ≤1.53 | Reference |  |  |
| >1.53 | 1.310 | 0.774-2.217 | 0.314 |
| **Loco-regional relapse–free survival** | | | |
| Treatment |  |  |  |
| CCRT | Reference |  |  |
| NTZ-CCRT | 0.452 | 0.190-1.075 | 0.072 |
| Gender |  |  |  |
| Male | Reference |  |  |
| Female | 0.294 | 0.069-1.242 | 0.096 |
| Age |  |  |  |
| ≤53 | Reference |  |  |
| >53 | 1.714 | 0.778-3.779 | 0.181 |
| Tumor stage |  |  |  |
| T1-T2 | Reference |  |  |
| T3-T4 | 1.723 | 0.799-3.716 | 0.166 |
| Node stage |  |  |  |
| N0-N1 | Reference |  |  |
| N2-N3 | 2.289 | 0.541-9.687 | 0.261 |
| LDH |  |  |  |
| ≤250 | Reference |  |  |
| >250 | 0.045 | 0.000-49.269 | 0.385 |
| hsCRP |  |  |  |
| ≤1.53 | Reference |  |  |
| >1.53 | 1.253 | 0.579-2.711 | 0.567 |

HR: Hazard ratio CI: confidence interval

Table S 2 **Multivariate Analysis of Prognostic Factors in NPC Patients.**

|  | HR | CI (95%) | P value |
| --- | --- | --- | --- |
| **Overall survival** | | | |
| Treatment |  |  |  |
| CCRT | Reference |  |  |
| NTZ-CCRT | 0.367 | 0.167-0.808 | 0.013 |
| Gender |  |  |  |
| Male | Reference |  |  |
| Female | 0.155 | 0.037-0.650 | 0.011 |
| Age |  |  |  |
| ≤53 | Reference |  |  |
| >53 | 1.731 | 0.885-3.386 | 0.109 |
| Tumor stage |  |  |  |
| T1-T2 | Reference |  |  |
| T3-T4 | 1.552 | 0.822-2.932 | 0.175 |
| Node stage |  |  |  |
| N0-N1 | Reference |  |  |
| N2-N3 | 2.474 | 0.754-8.117 | 0.135 |
| LDH |  |  |  |
| ≤250 | Reference |  |  |
| >250 | 5.170 | 2.125-12.580 | ﹤0.001 |
| hsCRP |  |  |  |
| ≤1.53 | Reference |  |  |
| >1.53 | 1.478 | 0.768-2.843 | 0.242 |
| **Disease–free survival** | | | |
| Treatment |  |  |  |
| CCRT | Reference |  |  |
| NTZ-CCRT | 0.536 | 0.312-0.919 | 0.023 |
| Gender |  |  |  |
| Male | Reference |  |  |
| Female | 0.440 | 0.207-0.931 | 0.032 |
| Age |  |  |  |
| ≤53 | Reference |  |  |
| >53 | 1.392 | 0.844-2.294 | 0.195 |
| Tumor stage |  |  |  |
| T1-T2 | Reference |  |  |
| T3-T4 | 1.734 | 1.065-2.825 | 0.027 |
| Node stage |  |  |  |
| N0-N1 | Reference |  |  |
| N2-N3 | 2.474 | 0.989-6.192 | 0.053 |
| LDH |  |  |  |
| ≤250 | Reference |  |  |
| >250 | 2.421 | 1.027-5.707 | 0.043 |
| hsCRP |  |  |  |
| ≤1.53 | Reference |  |  |
| >1.53 | 1.128 | 0.688-1.849 | 0.632 |
| **Distant metastasis-free survival** | | | |
| Treatment |  |  |  |
| CCRT | Reference |  |  |
| NTZ-CCRT | 0.622 | 0.349-1.108 | 0.107 |
| Gender |  |  |  |
| Male | Reference |  |  |
| Female | 0.449 | 0.201-1.002 | 0.051 |
| Age |  |  |  |
| ≤53 | Reference |  |  |
| >53 | 1.918 | 1.098-3.350 | 0.022 |
| Tumor stage |  |  |  |
| T1-T2 | Reference |  |  |
| T3-T4 | 2.000 | 1.176-3.403 | 0.011 |
| Node stage |  |  |  |
| N0-N1 | Reference |  |  |
| N2-N3 | 2.161 | 0.858-5.446 | 0.102 |
| LDH |  |  |  |
| ≤250 | Reference |  |  |
| >250 | 2.997 | 1.260-7.131 | 0.013 |
| hsCRP |  |  |  |
| ≤1.53 | Reference |  |  |
| >1.53 | 1.063 | 0.621-1.818 | 0.824 |
| **Loco-regional relapse–free survival** | | | |
| Treatment |  |  |  |
| CCRT | Reference |  |  |
| NTZ-CCRT | 0.488 | 0.203-1.174 | 0.109 |
| Gender |  |  |  |
| Male | Reference |  |  |
| Female | 0.317 | 0.075-1.343 | 0.119 |
| Age |  |  |  |
| ≤53 | Reference |  |  |
| >53 | 1.516 | 0.672-3.423 | 0.316 |
| Tumor stage |  |  |  |
| T1-T2 | Reference |  |  |
| T3-T4 | 1.673 | 0.768-3.642 | 0.195 |
| Node stage |  |  |  |
| N0-N1 | Reference |  |  |
| N2-N3 | 2.272 | 0.530-9.737 | 0.269 |
| LDH |  |  |  |
| ≤250 | Reference |  |  |
| >250 | 0.000 | 0.000 | 0.980 |
| hsCRP |  |  |  |
| ≤1.53 | Reference |  |  |
| >1.53 | 0.998 | 0.452-2.204 | 0.996 |

HR: Hazard ratio CI: confidence interval
